# Video-based learning of coping strategies for common errors improves laparoscopy training—a randomized study

**DOI:** 10.1007/s00464-023-09969-w

**Published:** 2023-03-21

**Authors:** F. Lang, A. S. Gerhäuser, C. Wild, E. Wennberg, M. W. Schmidt, M. Wagner, B. P. Müller-Stich, F. Nickel

**Affiliations:** 1 Department of General, Visceral, and Transplantation Surgery, Heidelberg University Hospital, Im Neuenheimer Feld 420, 69120 Heidelberg, Germany; 2 Department of Gynecology and Obstetrics, University Medical Center of Johannes Gutenberg University, Mainz, Germany

**Keywords:** Laparoscopy, Minimally invasive surgery, Surgical training, Coping model, Mastery model, Knot tying

## Abstract

**Aims:**

The aim of this study was to investigate whether shifting the focus to solution orientation and developing coping strategies for common errors could increase the efficiency of laparoscopic training and influence learning motivation. The concept of coping has been particularly defined by the psychologist Richard Lazarus [Lazarus and Folkman in Stress, appraisal, and coping, Springer publishing company, New York, 1984]. Based on this model, we examined the use of observational learning with a coping model for its effectiveness as a basic teaching model in laparoscopic training.

**Methods:**

55 laparoscopically naive medical students learned a standardized laparoscopic knot tying technique with video-based instructions. The control group was only offered a mastery video that showed the ideal technique and was free from mistakes. The intervention group was instructed on active error analysis and watched freely selectable videos of common errors including solution strategies (coping model) in addition to the mastery videos.

**Results:**

There was no statistically significant difference between the intervention and control groups for number of knot tying attempts until proficiency was reached (18.8 ± 5.5 vs. 21.3 ± 6.5, *p* = 0.142). However, there was a significantly higher fraction of knots achieving technical proficiency in the intervention group after first use of the coping model (0.7 ± 0.1 vs. 0.6 ± 0.2, *p* = 0.026). Additionally, the proportion of blinded attempts that met the criteria for technical proficiency was significantly higher for the intervention group at 60.9% vs. 38.0% in control group (*p* = 0.021). The motivational subscore “interest” of the validated score on current motivation (QCM) was significantly higher for the intervention group (*p* = 0.032), as well as subjective learning benefit (*p* = 0.002) and error awareness (*p* < 0.001).

**Conclusion:**

Using video-based learning of coping strategies for common errors improves learning motivation and understanding of the technique with a significant difference in its qualitative implementation in laparoscopy training. The ability to think in a solution-oriented, independent way is necessary in surgery in order to recognize and adequately deal with technical difficulties and complications.

**Supplementary Information:**

The online version contains supplementary material available at 10.1007/s00464-023-09969-w.

Laparoscopic suturing and knot tying is a complex skill to learn and is a key competence for many basic and advanced laparoscopic surgical procedures [3, 4]. Proficiency in laparoscopic suturing and tying of knots is often an essential surgical step or very important as part of complication management [5]. To ensure optimal outcome and patient safety, a safe, reliable, and rapid performance is necessary, as well as a high-quality level of knots [6]. Laparoscopic suturing and knot tying requires an appropriate amount of training until an appropriate level of competence is achieved [7–12]. Optimal laparoscopic knot training has been shown to be dependent on several factors: goal orientation, sensitive and objective performance criteria, adequate instruction and feedback, objective assessment, motivation, resources, and manpower [13]. Despite technical advances, the difficulty of laparoscopic knot tying challenges the wider application of minimally invasive surgery (MIS). Further development of training programs that exercise laparoscopic suturing at a high level is essential [4, 14].

A widely used instructional approach in laparoscopy training is 1:1 instruction and supervision by an expert [15]. Nevertheless, qualified and experienced instructors are a limited resource, and interindividual dynamics can also negatively influence training success [16]. Thus, there is a need for training opportunities that do not require the presence of an expert. The principle of video instruction and analysis has now become established for this purpose. As a low-cost and simple digital medium, videos are used in e-learning in multimodal training concepts [17–20]. Xeroulis et al. have shown that for learning laparoscopic knot tying, computer-based video instruction can be equivalently successful to instruction and feedback from an expert and can be independently sustained [4, 21].

A precondition for proficiency-based training is a standardized assessment. In order to measure learning progress, performance should be assessed using objective, standardized criteria in addition to measured training time and number of attempts [21–26]. The “Objective Structured Assessments of Technical Skills” (OSATS) score is composed of a global as well as a procedure-specific checklist and is used today as a standard for the evaluation of laparoscopic skills [27–29]. In addition to evaluating individual performance, this allows assignment to a competency level, i.e., a defined level of experience, but also specific and standardized objective feedback and definition of target criteria as a level of performance to aim for in a training session (1).

Principles of self-regulated learning (SRL) continue to become more common in medical education. This encourages the ability to continually evaluate and adjust one’s strategies and behaviors to optimize learning and performance [30]. For use within structured training programs, this approach appears promising [31]. Psychologist Richard Lazarus defined the concept of coping as part of his transactional stress model [1]. Based on this model, we examined the use of observational learning with a coping model for its effectiveness as a basic teaching model in laparoscopic training. In the following, a learning model in which common errors are made and appropriate coping strategies are demonstrated is called a “coping model.” This contrasts with error-free, fluent performance—literally “mastering” the task demonstrated. This mode of demonstration is called the “mastery model” in the following.

The aim of this study was to improve laparoscopy training by video-based learning of coping strategies for common errors as a pragmatic, efficient, and cost-effective learning approach in the concept of video instruction and self-regulated learning [2].

## Materials and methods

### Setting and participants

This study was conducted as part of a voluntary elective course for medical students in the clinical part of their studies at Heidelberg University. This study took place in the training center for MIS of the Department of General, Visceral and Transplantation Surgery at Heidelberg University Hospital. Only laparoscopically naïve students, i.e., those who had never previously participated in a laparoscopic training course and had less than two hours of laparoscopic experience, were included. They received a standardized, 4-h basic skill training on box and virtual reality (VR) trainers. All participants were informed of the duration, procedures, methods, and objectives of the study before written informed consent was obtained [2].

Sample size was calculated based on data from a pilot study with a comparable setting (*n* = 8). The average number of knot tying attempts until reaching the specified proficiency level was 16.3 ± 4.7 for the intervention group (mastery and coping model) and 28 ± 3.6 for the control group (mastery model only) of the pilot study. The difference was detected at a two-sided significance level *α* = 0.05 and a power of 1 − *β* = 0.8 with a group size of at least 3 participants per group. For meaningful results and to account for drop-outs, we planned to recruit a total of 60 participants [2].

All participants were randomized in a 1:1 ratio and assigned to an intervention or control group. Randomization was performed with a block size of 12 using an online tool (http://www.randomizer.org) by an independent staff member who was not otherwise involved in the design, conduct, or analysis of the study. Randomization results were kept in sealed, opaque, and sequentially numbered envelopes until students were assigned by the principal coordinator.

In each case, participant characteristics were recorded before training. To become familiar with the laparoscopic setting, all participants underwent the same basic training. In addition, they were asked to answer the validated questionnaires (Questionnaire on Current Motivation (QCM), self-efficacy expectations scale (ASKU), self-assessment of satisfaction with own performance) for further participant characterization [32–35].

To ensure consistent video-based instruction in the performance of laparoscopic knot tying on the box trainer using C-loop technique, all study participants were shown an error-free instructional video as a mastery model three times [10]. Subsequently, the first two knot attempts were performed and evaluated live by a trained tutor using checklists required for the level of performance to be achieved (proficiency level). The knots had to be performed independently without further instruction from the tutor.

The checklists included the 5-point Likert scale for assessing knot quality [36] and the adapted OSATS checklist for laparoscopic knot tying [37]. Knot quality assessment evaluates whether the knot has visible gaps between stacked throws, is tight at base, only edges are opposed, and knot holds under tension. The adapted OSATS checklist for laparoscopic knot tying evaluates the needle delivery/load, suturing, and knot tying separately. In order to provide the subjects with the most realistic and objective assessment possible, only the Procedural Checklist was used, excluding items that were not assessable on the box trainer in the present setting (maximum achievable score of 13) [2].

The checklists were available as online questionnaires and each score was recorded. In addition, the time required for each knot was measured. The time limit per laparoscopic knot was 20 min. The proficiency criteria were achieved if at least 4 of 5 points in the Knot Quality Score, 11 of 13 points in the OSATS Procedural Checklist (corresponding to ≥ 80% of the maximum score), and a knot time of max 2:00 min (min:s) were obtained. This matches performance levels achieved by experienced surgeons [23]. The proficiency criteria had to be achieved in two consecutive attempts [2].

### Study design

**Fig. 1:**
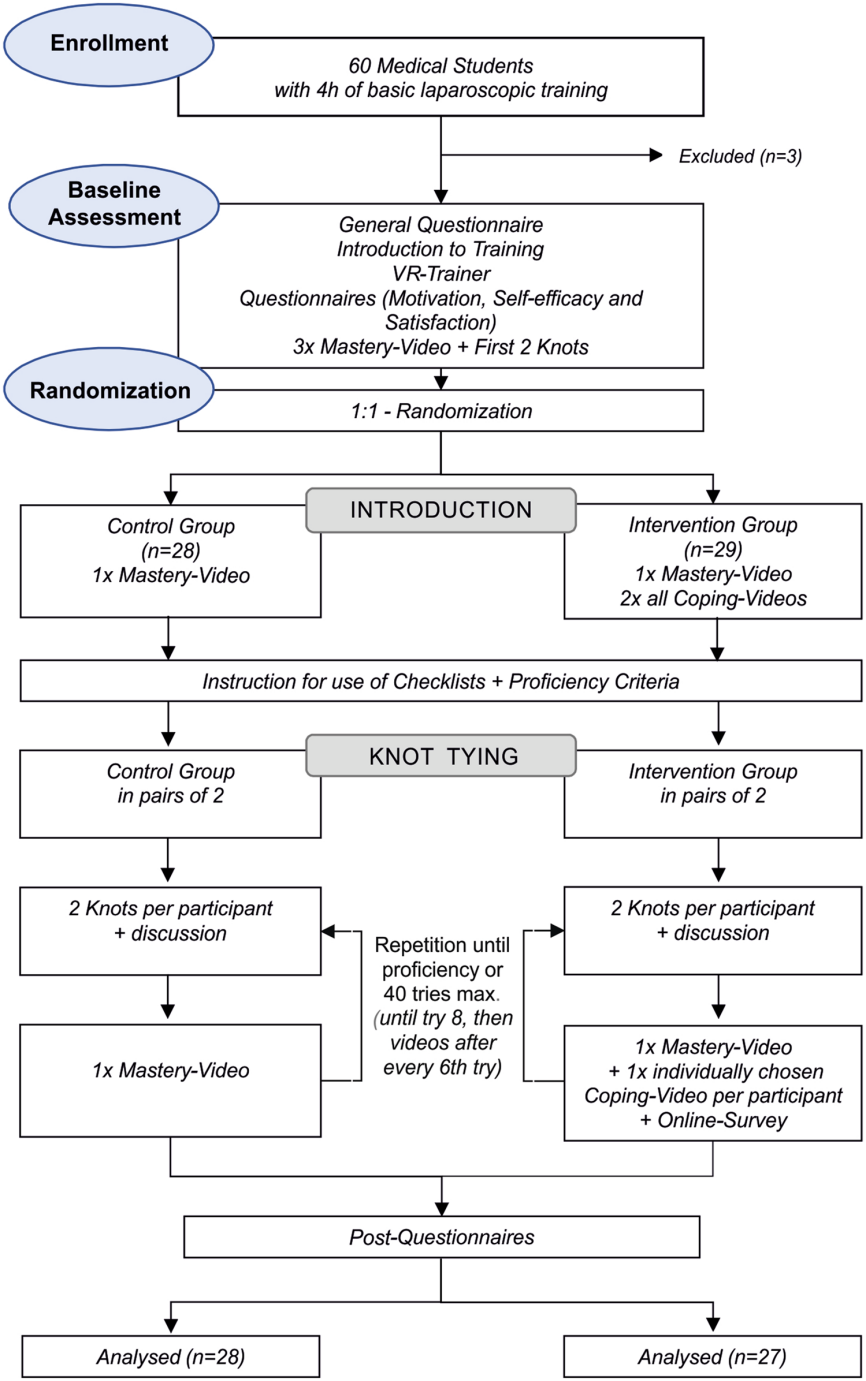
Flowchart of the study [2]

In this randomized controlled monocentric study, subjects were assigned to either the control or intervention group. Both groups learned laparoscopic suturing and knot tying in teams of two by video-based instructions. The intervention group was provided with laparoscopic suturing and knot tying instruction videos with various coping strategies for common errors (coping videos), as well as error-free instruction (mastery video). In addition, the intervention group was regularly instructed to actively analyze their own mistakes by means of a questionnaire (Fig. 1). The control group had only the mastery video available and did not fill out questionnaires about their own mistakes. Each subject was given their own tablet on which only their assigned videos were accessible. Participants learned the C-loop technique for laparoscopic suturing and knot tying. All participants practiced laparoscopic knot tying and suturing until reaching the specified proficiency level or up to a maximum of 40 attempts, watching the instructional videos after every two attempts at the beginning, depending on group assignment. Both training partners alternated after every two knots and evaluated the training partner using the checklists. A 2-min discussion of mutual evaluation followed in each case. After completing two knots per participant at a time, students were instructed to watch the mastery video once each. The intervention group received an individually selected coping video in addition to the mastery video. They were also asked to fill out an additional online questionnaire in which the selection of the coping video was justified [2]. Once participants rated one of their partner’s knots as proficient, a blinded rater was called in to directly assess the next knot to verify proficiency. Participants completed questionnaires on current motivation, self-efficacy, and satisfaction with their own performance after each training session and after reaching the required proficiency level.

Primary endpoint of the study represented the number of knot attempts and total training time until reaching proficiency criteria based on Knot Quality Score, the OSATS Procedural Checklist, and knot time of max 2:00 min. Secondary endpoints were parameters relevant to learning psychology–satisfaction with own expectation, self-efficacy expectation, and current motivation recorded by the validated questionnaires.

### Materials

The study was performed on a Szabo–Berci–Sackier box trainer and a standard laparoscopy tower (KARL STORZ GmbH & Co. KG, Tuttlingen, Germany). Participants sutured with two laparoscopic needle holders and a braided, absorbable Polysorb 3-0 suture with CV-25 cone ½ 22-mm needle (Medtronic Minimally Invasive Therapies TM, Minneapolis, MN, USA) shortened to 12 cm on a fixed silicone suture pad with predefined entry and exit points (Big Bite Medical GmbH, Heidelberg, Germany).

Coping videos used in this study were designed specifically for this purpose [2, 38]. The content of the coping videos was chosen to show common errors and solution strategies for laparoscopic knot tying [2, 39–44]. In addition, it was examined that the errors could also be represented in the technique checklists [2, 37]. Table 1 describes the content of the coping videos. Before conducting the study, aspects of validity evidence to collect validity evidence for the use of video-based learning of coping strategies for common errors in laparoscopy training by surgeons of different experience levels (novice, advanced, and expert in the field of laparoscopic knot tying) were investigated [2].

**Table 1:** Content and main focus of the coping videos [2, 38]

Coping video/addressed error	Solution strategy	OSATS-Item
1. ‟Needle load” (a) Improper needle load	Grasp needle 2/3 from tip and perpendicular to needle holder (i) Directly from the silicone pad (ii) Allow needle to stand up with light pressure into tissue with needle holder slightly open (iii) Rotate needle in front of camera to check orientation	1 2
(b) Poor positioning of the right needle holder	Grip to 1/3 from end of needle or to 2/3 from tip of needle	2
(c) Improper starting position of the needle	Pull the thread to the starting position (i) On the silicone pad (ii) With needle loosely gripped by non-dominant hand	1
2. ‟Tissue and instrument handling” (a) Traumatic tissue handling	Pierce at a 90° angle Pierce by slightly rotating the wrist to follow the shape of the needle	3 4
(b) The left instrument gets caught in the knot	Leave loose, tighten thread end on needle side first (to the back diagonally, so that thread slips off instrument)	7 8 9
(c) Interfering tissue parts	Assist with second instrument, guide tissue, create space, and bimanual work	10 11 15
3. “Tail length” (a) Tissue damage due to incision	Use of a needle holder as a deflection roller, with the thread being pulled through in the stitch exit direction	5
(b) Entanglement of the thread end that is too long in the knot	For pulling through, deflection principle, keep thread end as short as possible, grip in rear third (If necessary, loosen knot again first)	5
(c) Suture end too short	Carefully pull out the thread end again	4 5
4. ‟Needle orientation during knot tying” (a) Wrapping not possible	Regrasp or bring needle to appropriate angle (i) With rotational movement of the instrument/wrist (ii) With needle loosely grasped with non-dominant hand by pulling on thread Clean C-loop	7 8 9
(b) Winding in the wrong direction	Ensure correct positioning of needle end, C-loop and instruments	7 8 9
5. “Lift and drift” (a) Grasped needle leaves field of view	Put down needle, readjust thread	14
(b) Wrapping too high	Minimize the distance between tissue and instruments	7 8 9
(c) Thread slips repeatedly from the instrument	Understanding and moving instruments as a unit	11 15

In addition, validated questionnaires were used to survey learning psychology parameters, such as current motivation, satisfaction with own performance, and self-efficacy [2]. Satisfaction with own performance was assessed using a Likert scale with a range of 0–100, with 100 corresponding to maximum satisfaction [35]. Self-efficacy was assessed using the General Self-Efficacy Short Scale (ASKU) according to Beierlein et al. [33]. This is an instrument for recording individual competence expectations of being able to deal with difficulties and obstacles in daily life. The rating uses a Likert scale of 1–5, with an average of three items. The Questionnaire on Current Motivation (QCM) measures four components specific to the learning and performance situation with 18 items on a Likert scale of 1–7: Mean values result for the categories challenge, interest, probability of success, and anxiety [34].

### Ethical considerations

The study protocol was approved by the institutional ethics committee at Heidelberg Faculty of Medicine (S-436/2018) and informed consent was obtained from every participant.

### Statistical analysis

All continuous data are presented as mean values with standard deviation and were compared using Mann–Whitney *U* test to determine the differences between the groups. A double-sided *p* value of < 0.05 was considered statistically significant. All calculations were carried out using SPSS Software (Version 22.0, IBM SPSS Inc., Chicago, Illinois, USA).

## Results

A total of 60 participants were recruited for the study. 55 were included in the analysis. Two participants did not meet the inclusion criteria. Three additional participants dropped out during the study and thus were not analyzed.

Both groups showed similar demographic distribution (Table 2). There was no significant difference between the control group and the intervention group in the baseline tasks based on VR Score [45] (68.5 ± 20.0 vs. 73.5 ± 16.7; *p* = 0.058) [2].

**Table 2:** Baseline characteristics

		Control group (*n* = 28)	Intervention group (*n* = 27)
Age		23.9 ± 3.4	24.2 ± 3.5
Gender	Male	11 (39.3%)	15 (55.6%)
	Female	17 (60.7%)	12 (44.4%)
Dominant hand	Right	27 (96.4%)	24 (88.9%)
	Left	1 (3.6%)	3 (11.1%)
Regular video games		9 (32.1%)	10 (37.0%)
Interest in laparoscopic surgery [score, 0–100]		81.3 ± 17.5	81.5 ± 21.5

Mean ± sd

Regarding pre-test data collected in the beginning of the study with the baseline questionnaires for satisfaction with own performance, self-efficacy as well as the four components of the questionnaire current motivation, no difference was seen between the two groups (Table 3).

**Table 3:** Baseline results of parameters of psychology of learning

	Control group (*n* = 28)	Intervention group (*n* = 27)	*p*-value
Satisfaction with own performance [0–100]	66.7 ± 17.2	65.9 ± 13.9	0.713
ASKU (self-efficacy)	3.8 ± 0.3	3.8 ± 0.5	0.782
Challenge (QCM)	5.2 ± 0.7	5.3 ± 0.9	0.426
Interest (QCM)	5.2 ± 0.6	5.3 ± 0.8	0.261
Probability of success (QCM)	3.5 ± 0.5	3.3 ± 0.5	0.096
Anxiety (QCM)	2.9 ± 0.9	2.7 ± 1.2	0.288

Satisfaction (using a Likert scale with a range of 0–100, with 100 corresponding to maximum satisfaction), Self-Efficacy Short Scale (ASKU) (using a Likert scale of 1(does not apply at all)–5(applies completely)), and Questionnaire on Current Motivation (QCM) (measuring four components specific to the learning and performance situation with 18 items on a Likert scale of 1(does not apply at all)–7(applies completely))

Mean ± sd, * significant for *p* < 0.05, Mann–Whitney *U* test

At pre-test, there was no difference between the two groups for the OSATS procedural checklist, the time per knot, and the knot quality score (Table 4) [2].

**Table 4:** Baseline knot attempts

	Control group	Intervention group	*p*-value
Total score [procedural checklist, max. 13]	4.6 ± 2.2	5.3 ± 2.1	0.123
Time [s]	529.2 ± 280.7	532.1 ± 309.6	0.647
Total score [knot quality score, max. 5]	2.3 ± 1.5	2.6 ± 1.7	0.322

Mean ± sd, * significant for *p* < 0.05, Mann–Whitney *U* test

Video-based learning of coping strategies for common errors did not result in a significant difference between the intervention and control group in terms of total number of knot attempts until proficiency level in this study (18.8 ± 5.5 vs. 21.3 ± 6.5; *p* = 0.142). The total training time to proficiency did not show a significant difference between groups (4437.5 ± 1819.9 s vs. 4897.9 ± 1767.8 s, *p* = 0.297).

**Fig. 2:**
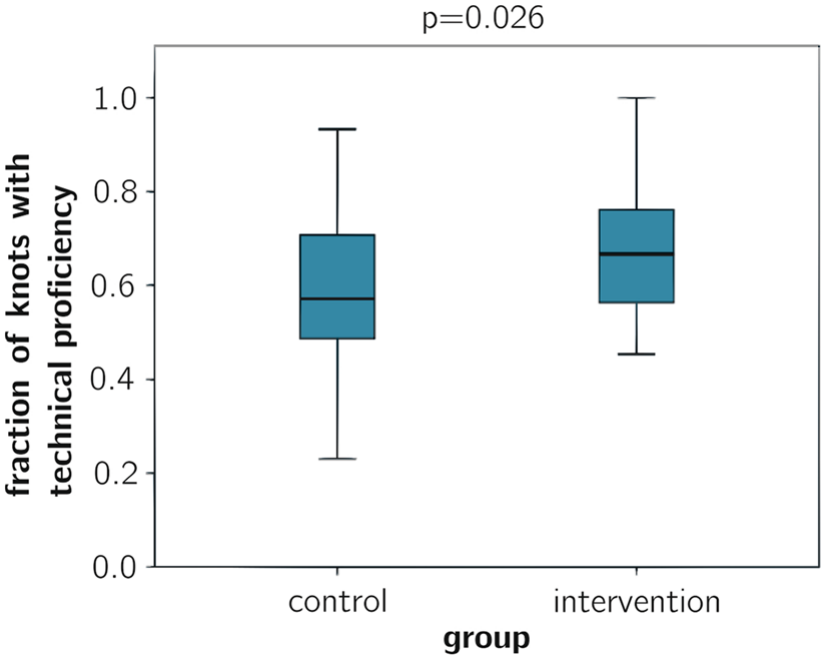
Proportion of knots achieving technical proficiency from the first intervention onward, *significant for *p* < 0.05, Mann–Whitney *U* test [2]

There was a significantly higher proportion for the number of technically successful knots after the first intervention (attempt 5) for the intervention group than for the control group (0.7 ± 0.1 vs. 0.6 ± 0.2; *p* = 0.026, Fig. 2). At this time, participants in the intervention group first selected a coping video. “Technical Proficiency” excludes time, so includes only the OSATS Procedural Score and the Knot Quality Score [2].

Looking at the knot attempts assessed by a blinded rater (proficiency attempts as well as individual samples), it is noticeable that the proportion of blinded attempts that met the criteria for technical proficiency (i.e., only failed to meet the time limit) was significantly higher for the intervention group at 60.9% vs. 38.0% in the control group (*p* = 0.021). Comparing scores on each item of the OSATS Procedural Checklist, the control group had more problems implementing the technique as specified in 10 of 13 items [2].

In the final survey (after reaching proficiency level), there was no difference in satisfaction with own performance. The self-efficacy short scale as secondary endpoint also showed no significant difference between the groups. The probability of success, challenge, and anxiety components of current motivation showed no difference. Interest was significantly higher for the intervention group (Table 5).

**Table 5:** Secondary endpoints (satisfaction, ASKU, QCM)

	Control group	Intervention group	*p*-value
Satisfaction with own performance [0–100]	82.0 ± 14.5	80.9 ± 17.3	1.000
ASKU (self-efficacy) [rawscore, 1–5]	4.0 ± 0.6	4.1 ± 0.6	0.448
Challenge (QCM) [rawscore , 1–7]	5.5 ± 0.8	5.7 ± 1.0	0.139
Interest (QCM) [rawscore , 1–7]	4.8 ± 1.4	5.5 ± 1.1	0.032*
Probability of success (QCM) [rawscore , 1–7]	3.3 ± 0.6	3.2 ± 0.6	0.721
Anxiety (QCM) [rawscore , 1–7]	2.9 ± 1.4	2.9 ± 1.6	0.946

Mean ± sd, * significant for *p* < 0.05, Mann–Whitney *U* test

All participants were asked to complete the questionnaires on current motivation, satisfaction with own performance, and self-efficacy on a frequent interval during the study [2]. The training progress of 0% corresponds to the baseline results and 100% to the final survey results. Interest increased over the laparoscopic knot training in the intervention group and decreased significantly in the control group, reaching a low point in the last quarter of training progress. The curves differ considerably here, and the final value for interest was significantly higher for the intervention group. The challenge posed by the laparoscopic knot was rated higher by the intervention group overall and during the course than by the control group. The challenge was maximal in the first half of training for the control group. For both groups, it increased over the entire training period, reaching a maximum in the intervention group and a minimum in the control group simultaneously at 76%–99% training progress. The probability of success was estimated higher by the control group than by the intervention group. In both groups it decreased overall in the course. For the training progress of 51%–75% it reached a maximum in the control group and in the following point of comparison a minimum in the intervention group [2].

The intervention group completed an online survey after each use of the coping videos to analyze errors and the usefulness of the coping videos [2]. The total number of surveys was 111.

Four items most frequently rated as problematic on the OSATS procedure score were second winding of the knot (item 8; 24.3% of surveys), correct guidance of the needle when piercing the tissue (item 4; 22.5%), picking up the needle (item 1; 21.6%), and first winding of the knot (item 7; 19.8%).

81.1% of the participants from the intervention group rated the coping videos as helpful. The control group did not perform any such error analysis, because they only had access to the mastery video [2].

After the end of the study, the subjects were asked about aspects of their subjective learning success (Table 6). No difference was found in the ability to concentrate on the instructional videos. Participants in the intervention group agreed with an increased interest in surgery as a result of video-based learning of coping strategies. They felt significantly better prepared than the control group. Both the usefulness of the instructional videos and the learning gains were rated significantly better by the intervention group than the control group. The subjects in the intervention group also rated themselves as significantly more confident in recognizing their own mistakes. In contrast, the control group reported significantly greater difficulties in identifying technical errors. Participants in the control group felt significantly less prepared for the intraoperative use of the learned knot tying technique. The subjective learning success was significantly greater overall for the intervention group (Table 6) [2].

**Table 6:** Subjective learning success

Approval [score 0–100]	Control group	Intervention group	*p*-value
Increase interest in surgery	76.8 ± 27.2	88.9 ± 20.0	0.046*
Good preparation through video clips	33.9 ± 22.8	50.9 ± 26.4	0.010*
Usefulness of video(s)	65.7 ± 28.6	86.3 ± 20.2	0.002*
Learning gain	63.6 ± 28.8	84.4 ± 21.4	0.002*
Ability to concentrate on the video(s)	81.4 ± 13.8	76.7 ± 26.3	0.965
Error awareness	49.1 ± 29.3	76.9 ± 26.8	0.001*
Difficulties error recognition	60.7 ± 29.2	31.5 ± 26.5	< 0.001*
Preparation for intraoperative laparoscopic knot tying	50.0 ± 24.5	75.9 ± 22.4	< 0.001*

Mean ± standard deviation, * significant for *p* < 0.05, Mann–Whitney *U* test

## Discussion

In the present randomized controlled study, the use of video-based learning of coping strategies for common errors in laparoscopy training [23] did not significantly reduce training time to proficiency. Nevertheless, the use of coping videos significantly enhanced the quality of knots by increasing the proportion of knots reaching technical proficiency. In addition, the intervention group scored higher for the motivation factor interest after completing the study and rated their subjective learning success better. The participants felt significantly better prepared for intraoperative use of the learned technique and were more confident in recognizing their own errors. In contrast, the control group reported greater difficulty in recognizing technical errors.

Video-based learning of coping strategies for common errors did not reduce total training time but significantly improved technical implementation of laparoscopic suturing and knot tying in laparoscopy training [2]. It seems possible that the control group was more focused on time and may have lacked vigilance against errors [2]. Smith et al. stated as early as 2001 that duration alone does not adequately reflect surgical events and circumstances. The assessment of laparoscopic skills should therefore be expanded to include at least the factors of precision and accuracy [48]. Overall, time alone is a questionable measure for assessing the quality of a surgical procedure. Ritter et al. were especially not able to demonstrate a correlation between the time required for performance and laparoscopic knot tying quality [49].

The motivational component interest was significantly higher using video-based learning of coping strategies for common errors. With regard to changes in motivation to learn with coping strategies, numerous variables are related to the onset and maintenance of readiness to learn. These include interest, perceived relevance, goal orientation, and self-efficacy [52]. In contrast to our study, Dempsey and Kauffmann found an increase in satisfaction and self-efficacy-expectancy using a coping model [35]. The control group rated their probability of success higher but this decreased sharply as the training progressed. The intervention group achieved technical proficiency on a higher proportion of knot attempts. One possible interpretation is that the coping videos led to a more realistic assessment of the demands of laparoscopic knot tying [2]. Surgeons who perform minimally invasive procedures have a particular obligation to accurately assess their own competences and learning needs in this area. It is desirable in this setting that surgeons develop and maintain accurate self-efficacy-expectations [53]. Artino et al. determined that low-performing medical students exhibited deficiencies in motivational components of self-regulated learning. These again included low self-efficacy-expectancy and low estimated task relevance. These findings highlight the need for individual support strategies to improve these components [54]. Margolis and Mccabe recommended active coping experiences for this purpose as in model learning [55]. Regarding motivation as a dependent variable in medical education, e.g., autonomy and the perceived own ability to act (in the sense of self-efficacy-expectation), were mentioned repeatedly as factors that are indispensable for intrinsic motivation [56]. In the present training approach, these aspects were addressed as follows. In coping videos, a learning model was used to demonstrate that mistakes happen and why they should be avoided. Coping videos demonstrated a range of coping strategies to expand the viewers’ options for action, and in the intervention group, subjects were able to determine which coping video to watch after actively analyzing their own errors (autonomy). In addition to the increase in learning motivation, a positive influence on the realistic assessment of the demands of laparoscopic knot tying has also been observed that is ultimately a prerequisite for the necessary realistic assessment of one’s own abilities [53].

Considering technical understanding and subjective confidence as prerequisites for surgical work, this study showed that subjective learning success increased significantly in all parameters using video-based learning of coping strategies [2]. The participants felt significantly better prepared for intraoperative use of learned techniques and rated learning gain and usefulness of instructional videos significantly higher. In particular, error awareness was greatly increased by coping videos. The competence to recognize one’s own mistakes is especially important, as beginners in particular tend to overestimate or underestimate their technical abilities [57, 58]. In this context, Hu et al. could not find any correlation between subjects’ confidence and speed or proficiency [58]. In contrast, Alameddine et al. showed that discrepancies in the assessment of surgical competencies and sense of safety by supervisors and the learners themselves complicated communication in teaching and learning processes [59]. Accurate self-assessment is critical for both promoting self-efficacy and avoiding overconfidence [58]. This competence is indispensable to avoid wrong decisions that can have critical consequences, especially for the patient. Surgeons who misjudge their capabilities may perform procedures for which they are not qualified, refer cases to less-qualified surgeons or decide to perform alternative procedures [53]. Technical understanding and subjective safety, in particular error awareness, were significantly improved using video-based learning of coping strategies for common errors in laparoscopic training. Technical understanding and subjective safety are prerequisites for a realistic assessment of one’s own abilities and limitations and are necessary for the conscious development of coping strategies. According to Quick et al. self-assessment becomes more realistic as the surgeon progresses in training [57]. Whether this effect can be accelerated by coping videos needs to be investigated in further studies with appropriate design and objective comparability. The use of video-based coping for errors should also be assessed more broadly in multimodal laparoscopy training on the one hand [60–64]. On the other hand, video-based coping could also be used to prepare for less common but potentially dangerous errors and their handling.

### Limitations

In contrast to the mastery video, the coping videos were only available from a right-handed perspective. The influence of this circumstance remains questionable, since the focus of the coping videos was on individual technical errors and not on the overall motion sequence. In addition, a total of only four left-handers participated in the study, three of whom were in the intervention group [2]. The training sessions took place at different days of week and times of day. In addition, the room temperature was sometimes very high during the study. All of these are possible confounding variables for subjects’ ability to concentrate and motivate. The parameters may have ultimately led to greater variability in performance. Subjects had changing training partners depending on the date, so individual competence, teamwork ability, and training progress could be influenced by varying degrees [62, 63, 65]. However, because instructors, personal, and environmental conditions also change in surgical training and in everyday professional life, these circumstances can also be considered a realistic condition. With the intention of recording the used parameters to be depicted as reliably as possible, only validated psychological questionnaires were used (QCM and ASKU). However, the individual items of these questionnaires are formulated in general terms, especially in ASKU, and were therefore only of limited significance for the specific parameters to be determined in this particular training setting. For example, the wording of the second of three items is “I can cope well with most problems on my own.” Phrases that inquire about the assessment of one’s own ability to cope with the laparoscopic knot technique (as a problem) would have allowed much more precise statements [2]. Nevertheless, the present randomized controlled study was able to elaborate the positive influence of video-based learning of coping strategies for common errors in laparoscopy training. With the present setting, it was possible to demonstrate a qualitatively improved technical implementation of laparoscopic suturing and knot tying in laparoscopy training. A number of psychological parameters such as learning motivation, interest in the task, and subjective confidence could also be investigated in more detail. Factors such as error awareness were more pronounced, especially using the coping strategy. These are all prerequisites for independent thinking, learning, and the development of problem-solving strategies, which are necessary for surgeons facing technical difficulties and complications in the operating room [2].

## Conclusion

The use of video-based training for coping strategies for common errors resulted in improved implementation of the knot tying technique, but the total training duration could not be reduced. The motivation to learn, especially the interest in the task, was increased. Understanding of the technique and subjective confidence in knot tying was increased, and error awareness was significantly greater for participants in the intervention group who learned with video-based training for common errors using the coping model. For the future, it will be interesting to see whether the use of coping models will not only positively change the learning condition but also show an improvement in real operations in particular, thus increasing patient safety.

## Supplementary Information

Below is the link to the electronic supplementary material.

Supplementary file1 (JPG 4043 KB)

Supplementary file2 (MP4 44711 KB)
