# Supplementary figures and images for: Video-based learning of coping strategies for common errors improves laparoscopy training—a randomized study
Source: Surg Endosc. 2023 Mar 21;37(5):4054–64. doi: 10.1007/s00464-023-09969-w (PMC10156798; doi:10.1007/s00464-023-09969-w)

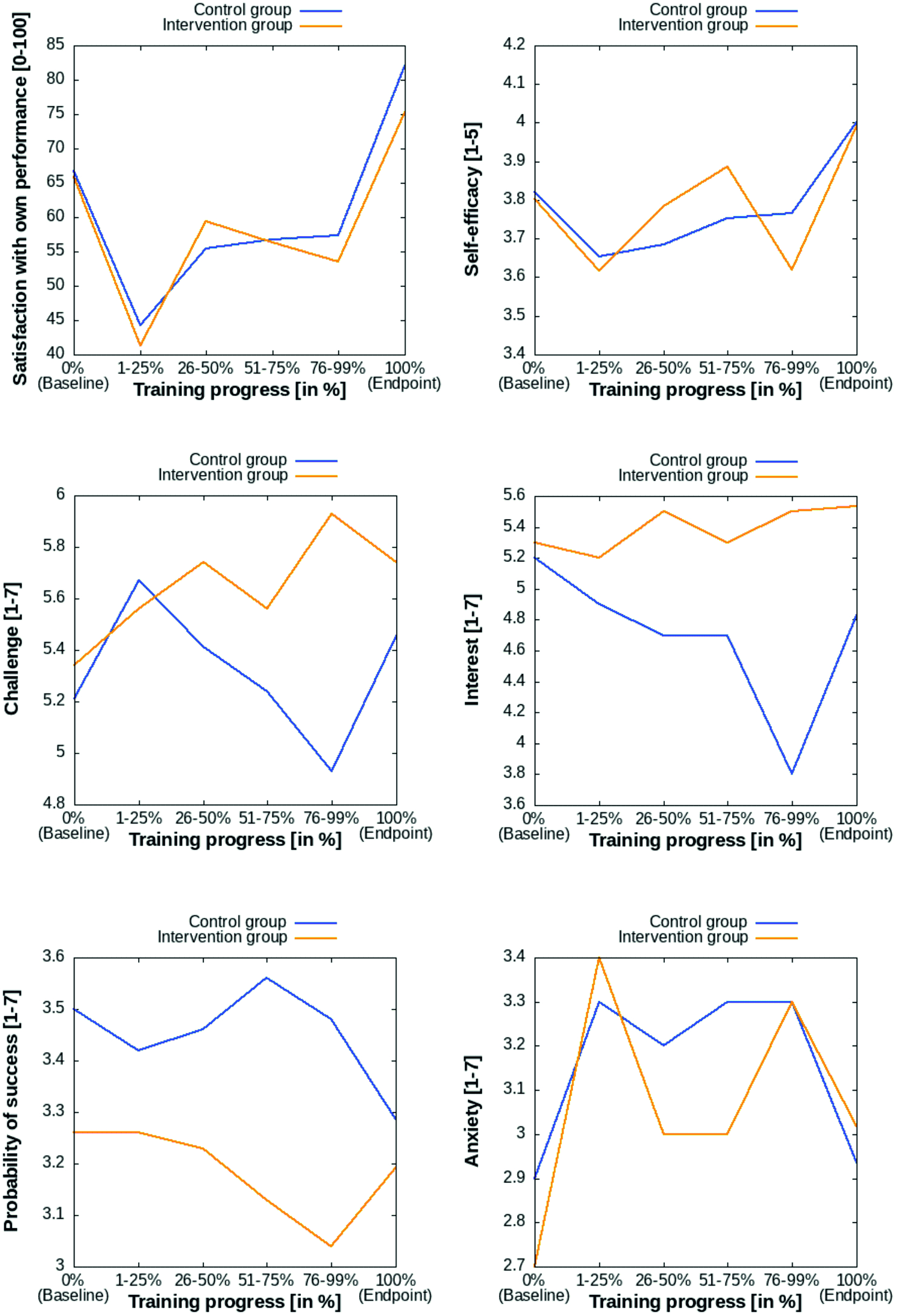

Supplement: Supplementary file 1 — Supplementary file1 (JPG 4043 KB) [file 464_2023_9969_MOESM1_ESM.jpg]
